# The anterolateral thigh flap for soft tissue reconstruction in patients with tongue squamous cell carcinoma

**DOI:** 10.1186/s12957-016-0972-8

**Published:** 2016-08-12

**Authors:** Xiang-Juan Tong, Zhan-Gui Tang, Zhen-Feng Shan, Xin-Cheng Guo

**Affiliations:** 1 Department of Oral and Maxillofacial Surgery, Xiangya Hospital, Central South University, Changsha, Hunan China; 2 Department of Oral and Maxillofacial Surgery, Xiangya Stomatological Hospital, Central South University, Changsha, Hunan China; 3 Department of Head and Neck Surgery, Hunan Provincial Tumor Hospital, Affiliated Tumor Hospital of Xiangya Medical School, Central South University, Changsha, Hunan China; 4 Department of Oral and Maxillofacial Surgery, Third Xiangya Hospital, Central South University, Changsha, Hunan China

**Keywords:** Oral cancer, Tongue squamous cell carcinoma, Anterolateral thigh flap, Reconstruction

## Abstract

**Background:**

Surgery remains the first choice of treatment for tongue cancer. Immediate reconstruction should be performed after wide resection of tumour. The aim of this study was to evaluate the anterolateral thigh flap for reconstruction of lingual defects.

**Methods:**

We report 39 consecutive oral tongue squamous cell carcinoma patients who underwent lingual reconstruction with the anterolateral thigh flap between 2009 and 2010.

**Results:**

The width of the skin island was 4 to 5 cm and the length of the skin island was 6 to 8 cm in 31 patients with T2 tumour, while the width of the skin island was 5.5 to 6 cm and the length of the skin island was 9 to 12 cm in 8 patients with T3/4 tumours. The all flap survival rate was 97.5 % in our series.

**Conclusions:**

We could obtain sufficient flap volume using the anterolateral thigh flap for tongue reconstruction. The single perforator-based anterolateral thigh flap is a good option for soft tissue reconstruction in patients with oral tongue squamous cell carcinoma.

## Background

Oral tongue squamous cell carcinoma (OTSCC) is the most common cancer diagnosed in the oral cavity comprising 25–40 % of oral carcinomas [1, 2], which demonstrates much more aggressive behaviour because of its unusual histologic makeup (rich lymphatic network and highly muscularized structure) [3]. OTSCC is thus more frequently associated with metastasis to draining lymph nodes than any other cancer of the oral cavity [4]. The major risk factor for OTSCC is chronic exposure of the oral mucosa to tobacco, alcohol and betel quid; they have a synergistic effect on carcinogenic development. Tongue submucous fibrosis and leukoplakia are also the most common premalignant lesions, and a betel quid chewing habit has usually been regarded as the main aetiology of submucous fibrosis [5, 6]. The reconstruction of a tongue defect is particularly challenging due to its comprehensive functions including articulation, deglutition and taste. Surgery remains the first choice of treatment for tongue cancer. Immediate reconstruction should be performed after wide resection of tumour [7]. The main aim is good function; aesthetic inside the mouth is secondary; and the main functions include articulation, deglutition and taste. Impairment of tongue function can severely affect quality of life. However, the restoration of the bulk, mobility and sensibility of the tongue also is one of the great challenges for surgeons. In the last 30 years, with the development of microsurgical reconstruction techniques, various free flaps have been described for extended lingual defect. In the last 20 years, the radial forearm flap has most commonly been used for reconstruction after hemiglossectomy.

However, the radial forearm flap sacrifices a major artery at the donor site and leaves a cosmetically unfavourable scar. In the last 10 years, the anterolateral thigh (ALT) flap has come into popular use. The ALT flap can supply a large amount of soft tissue with the possibility of flap thinning and its long pedicle, and surgery can generally be performed in a two-team approach with a low donor site morbidity [8, 9]. Furthermore, complex tongue defects involving the tongue and the floor of the mouth require accurate multiplanar configuration of flap design and tailoring. The flap design is the key point in both preserving mobility and providing neotongue bulk. The purpose of this study was to describe the ALT flap supplied by a single perforator for soft tissue reconstruction in 39 cases with tongue cancer and to evaluate the survival characteristics of this flap.

## Methods

**Fig. 1:**
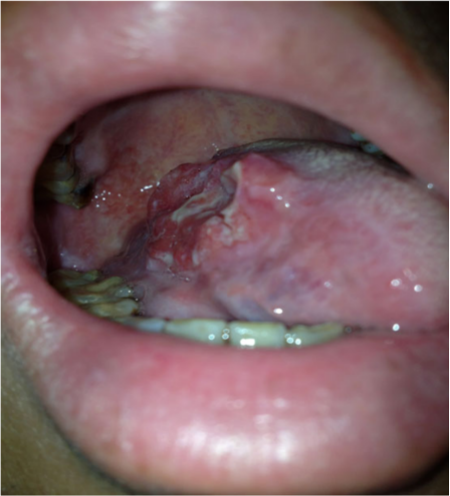
The carcinoma of right border of the tongue

Our study included 39 consecutive cases (30 males and 9 females) of biopsy-proved tongue squamous cell carcinoma and reconstructed by free ALT flap between 2009 and 2010 in our department (Fig. 1). The age groups ranged from 28 to 72 with a median age of 51. According to UICC classification (6th edition), there were 31 cases classified as T2, 3 as T3 and 5 as T4. The harvest of the flap, selection of the drainage vein and recipient vessel, microvascular anastomoses and postoperative management were performed as previously presented [10]. The flap was usually based on a single dominant perforator arising from a descending or oblique branch of the lateral circumflex femoral artery. Postoperatively, close monitoring of the exposed flaps in the first 72 h after surgery was performed hourly besides assessment and pinprick testing when colour, tactility, capillary refill, bleeding and appearance of the flap suggested a vascular problem. The frequency of flap monitoring was reduced to every 4 h thereafter until the first 7 days after surgery. The study was approved by the Institutional Review Board of Central South University, Hunan, China, and in our study, we always complied with the Helsinki Declaration.

## Results

**Fig. 2:**
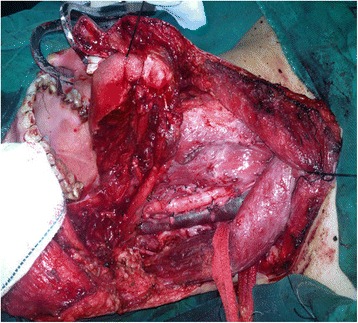
Partial glossectomy and functional neck dissection

**Fig. 3:**
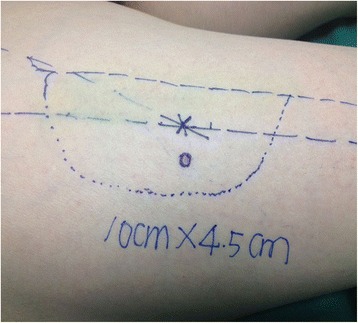
Preoperative markings of the stealth pattern

**Fig. 4:**
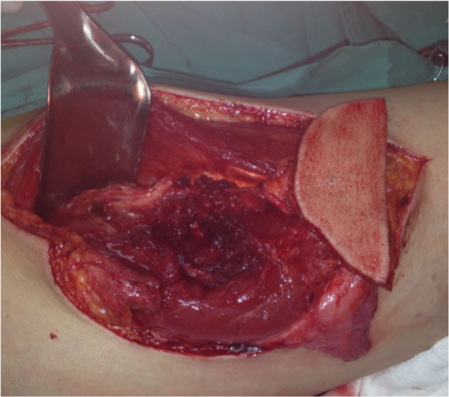
A harvested ALT flap

**Fig. 5:**
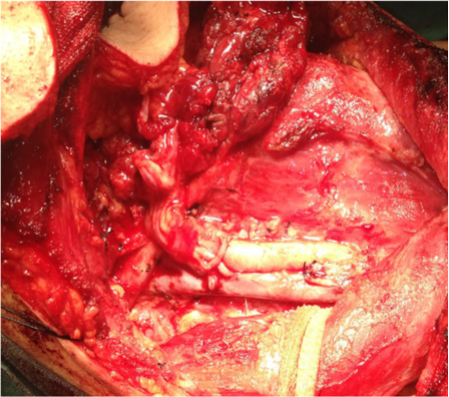
Anastomosis between the superior thyroid artery and the lateral circumflex femoral arteries matched in size

Primary tumour resection and functional neck dissection were indicated in all 39 cases. After, serial intraoperative frozen sections had been proved negative. Pathologic findings were as follows: 19 patients with stage II (T2N0M0); 10 patients with stage III (eight T2N1M0 and two T3N1M0); and 10 patients with stage IV (four T2N2M0, one T3N2M0, three T4N1M0 and two T4N2M0). Five patients with T4 underwent tracheostomy for 1 week. Postoperative patients were advised to return visit regularly at intervals of 3 months in the first year and thereafter once every 6 months to 1 year. All the 39 patients used nasogastric tubes for feeding after operations for 10 days. Characteristics of the 39 patients who underwent reconstruction of the tongue and the floor of the mouth with the ALT flap are listed in Table 1 and Fig. 2. All the flaps were harvested on a single dominant perforator (Figs. 3, 4 and 5). The width of the flap ranged 4 to 6 cm, with a mean width of 4.70 ± 0.61 cm. The length of the flap ranged 6 to 12 cm, with a mean length of 7.46 ± 1.79 cm. The size of the flap ranged 24 to 66 cm^2^, with a mean size of 35.94 ± 12.91 cm^2^. The width of the skin island was 4 to 5 cm and the length of the skin island was 6 to 8 cm in 31 patients with T2 tumour, while the width of the skin island was 5.5 to 6 cm and the length of the skin island was 9 to 12 cm in 8 patients with T3/4 tumours. The perforator was musculocutaneous in 36 patients, while septocutaneous in 3 patients. In patients with a musculocutaneous perforator, it was dissected through the muscle. The perforator was arising from the descending branch in 35 patients, while in 4 patients from the oblique branch of the lateral circumflex femoral artery.

**Table 1:** Patient and flap characteristics

Pt#	Age (year)	Gender	T class	Size	Size (cm^2^)	Perforators	Pedicle	Single pedicle	Complications
1	72	F	4	5.5 × 12	66	MC	D	Y	None
2	45	M	2	4 × 6	24	MC	D	Y	None
3	49	M	4	6 × 10	60	MC	D	Y	None
4	52	M	2	5 × 7.5	37.5	MC	D	Y	None
5	44	F	2	4 × 6	24	MC	O	Y	None
6	58	M	2	5 × 7	35	MC	D	Y	None
7	38	F	4	5.5 × 12	66	SC	D	Y	None
8	67	M	2	5 × 7	35	MC	D	Y	None
9	68	F	2	4 × 6	24	MC	D	Y	None
10	66	M	2	5 × 7.5	37.5	SC	D	Y	None
11	52	M	2	5 × 8	40	MC	O	Y	None
12	49	M	2	5 × 7	35	MC	D	Y	None
13	55	M	2	4 × 6	24	MC	D	Y	None
14	44	M	4	5.5 × 11	60.5	MC	O	Y	None
15	46	F	2	4 × 6	24	MC	D	Y	None
16	28	F	2	4 × 6	24	MC	D	Y	None
17	44	M	2	5 × 7	35	MC	D	Y	None
18	55	M	2	5 × 7	35	MC	D	Y	None
19	35	F	4	5.5 × 11	60.5	MC	O	Y	None
20	71	F	2	4 × 6	24	MC	D	Y	None
21	44	M	2	5 × 7	35	SC	D	Y	None
22	45	M	2	4 × 7	28	MC	D	Y	None
23	33	M	2	4 × 6	24	MC	D	Y	None
24	64	M	2	5 × 7	35	MC	D	Y	None
25	55	M	2	5 × 8	40	SC	D	Y	None
26	50	M	2	5 × 7	35	MC	O	Y	None
27	32	F	2	4 × 6	24	MC	D	Y	None
28	65	M	2	5 × 7	35	MC	D	Y	None
29	42	M	2	4 × 6	24	MC	D	Y	None
30	64	M	2	4 × 6	24	MC	O	Y	Infection
31	34	M	2	4 × 6	24	MC	D	Y	None
32	51	M	2	5 × 7	35	MC	D	Y	None
33	59	M	2	5 × 7	35	MC	D	Y	None
34	61	M	3	5.5 × 10	55	MC	D	Y	None
35	53	M	2	4 × 6	24	MC	D	Y	None
36	72	M	3	5.5 × 9	49.5	MC	D	Y	None
37	65	M	3	4.5 × 10	45	MC	D	Y	None
38	51	M	2	5 × 7	35	MC	D	Y	None
39	40	M	2	4 × 6	24	MC	D	Y	None

*MC* musculocut, *SC* septocut, *D* descending branch of lateral circumflex femoral artery, *O* oblique branch of lateral circumflex femoral artery

In all the cases, the defects involved the tongue and the floor of the mouth. In this series, ALT flap was used as a single flap to reconstruct these defects. The all flap survival rate was 97.5 % in our series. There was delayed loss of a flap due to infection leading to thrombosis in venous anastomosis on the sixth day after surgery, and in this case, the perforator was arising from the oblique branch, the terminal external diameter of which was 0.5 mm.

## Discussion

The ALT flap was first reported by Song et al. as a septocutaneous perforator-based flap [11], and it was found that the blood supply of the ALT flap is based on the septocutaneous or myocutaneous perforators or both [12]. Variations in the anatomy of the vascular pedicle and a difficult dissection technique initially led to a lack of popularity of this flap. Recently, the advantages of the ALT flap have been highlighted [13, 14]. In our study, we found that the ALT flap is relatively reliable and easy to raise, with a vascular pedicle of 8 cm or more in all cases. All flaps were anastomosed to the internal jugular vein and the superior thyroid artery without tension. The proportion of the flaps with septocutaneous perforators in our series was 10.3 %.We also found that in 34/39 cases (87.2 %), the perforators originated from the descending branch of the lateral circumflex femoral artery.

**Fig. 6:**
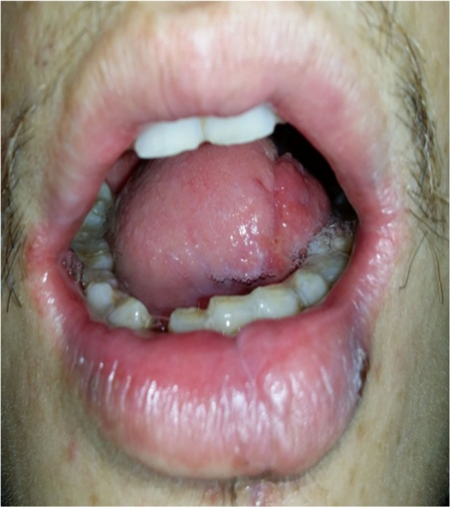
ALT flap post glossectomy

We have not experienced any problems with donor site morbidity, in any case in which all donor sites were closed directly. When selecting a flap for lingual reconstruction, the ALT flap is an excellent option with a more aesthetic donor site than its main rival, the radial forearm flap. The ALT flap decreases in size postoperatively partly through a reduction in the subcutaneous fat and partly because of fibrosis secondary to radiotherapy (Fig. 6).

In our study, there are several points about the ALT flap for lingual reconstruction that may be worthy of discussion. Firstly, it is safer to make the first incision relatively medial in the thigh and the midline is often a good choice. Secondly, it is important to complete the dissection of the perforator; musculocutaneous perforator dissection is not easy; many tiny branches arise from the lateral aspects to the muscle itself during dissection of an intramuscular perforator. Starting at the deep aspect of the fascia lata and working from distal to proximal, it is also safe to dissect a perforator only. Thirdly, it is useful to mark one aspect of a perforator for minimizing the possibility of twisting the perforator, which gives reassurance that the pedicle has not become twisted when it is mobilized and set in place. Fourthly, it is advantageous to delay the lateral skin incision in the thigh and to wait until after resection for accurate measurements of the required skin paddle and avoiding excess ischaemic time. Finally, the length of harvesting ALT flap ranges from 6 to 12 cm and the width from 4 to 6 cm for the tongue and the floor of the mouth reconstruction.

Our success rate in the ALT flap based on the single perforator for tongue reconstruction has been favourable. In our study, the microvascular patency is particularly good, and there was only one case of complete flap loss. In this case, the flap loss was after the sixth post operative day secondary to bacterial infection and vein anastomotic thrombosis; furthermore, the perforator aroused from the oblique branch of the lateral circumflex femoral artery with a vascular pedicle of only 8 cm, and the terminal external diameter of which was 0.5 mm, and we used the operating microscope in this case.

In our practice, adoption of the ALT flap has almost replaced the requirement for the rectus abdominis flaps and the radial forearm flaps in tongue reconstruction. The reduction of donor site morbidity, avoidance of the need for skin grafts and acceptability of the site of the resultant scar are major advantages over most alternative options. However, some glossal defects in obese patients in our study were reconstructed with an ALT flap, which showed excess bulk. Techniques to thin the ALT flap have been described but seem to be technically difficult.

## Conclusions

We could obtain a sufficient flap volume using the ALT flap for tongue reconstruction. The single perforator-based ALT flap is a good option for soft tissue reconstruction in patients with oral tongue squamous cell carcinoma.

## Abbreviations

ALT flap, anterolateral thigh flap; OTSCC, oral tongue squamous cell carcinoma; UICC, Union for International Cancer Control

## References

[1] Brown B, Barnes L, Mazariegos J, Taylor F, Johnson J, Wagnerr RL (1989). Prognostic factors in mobile tongue and floor of mouth carcinoma. Cancer.

[2] Krishna Rao SV, Mejia G, Roberts-Thomson K, Logan R (2013). Epidemiology of oral cancer in Asia in the past decade—an update (2000–2012). Asian Pac J Cancer Prev.

[3] Lim MS (2006). Re: correlational of oral tongue cancer inversion with matrix metalloproteinases (MMPs) and vascular endothelial growth factor (VEGF) expression, by Kim S-H, Cho NH, Kim K, et al. J Surg Oncol.

[4] Sano D, Myers JN (2007). Metastasis of squamous cell carcinoma of the oral tongue. Cancer Metastasis Rev.

[5] Sheth SH, Johnson DE, Kensler TW, Bauman JE (2015). Chemoprevention targets for tobacco-related head and neck cancer: past lessons and future directions. Oral Oncol.

[6] Zhang Y, Wang R, Miao L, Zhu L, Jiang H, Yuan H (2015). Different levels in alcohol and tobacco consumption in head and neck cancer patients from 1957 to 2013. PLoS One.

[7] Wang X, Yan G, Zhang G, Li J, Liu J, Zhang Y (2013). Functional tongue reconstruction with the anterolateral thigh flap. World J Surg Oncol.

[8] Koshima I, Fukuda H, Yamamoto H, Moriguchi T, Soeda S, Ohta S (1993). Free anterolateral thigh flaps for reconstruction of head and neck defects. Plast Reconstr Surg.

[9] Bussu F, Salgarello M, Adesi LB (2011). Oral cavity defect reconstruction using anterolateral thigh free flaps. B-ENT.

[10] Wei FC, Jain V, Celik N (2002). Have we found an ideal soft-tissue flap? An experience with 672 anterolateral thigh flaps. Plast Reconstr Surg.

[11] Song YG, Chen GZ, Song YL (1984). The free thigh flap: a new free flap concept based on the septocutaneous artery. Br J Plast Surg.

[12] Kimata Y, Uchiyama K, Ebihara S, Nakatsuka T, Harii K (1998). Anatomic variations and technical problems of the anterolateral thigh flap: a report of 74 cases. Plast Reconstr Surg.

[13] Chana JS, Fc W (2004). A review of the advantages of the anterolateral thigh flap in head and neck reconstruction. Br J Plast Surg.

[14] Shaw RJ, Batstone MD, Blackburn TK (2010). The anterolateral thigh flap in head and neck reconstruction: “pearls and pitfalls”. Br J Oral Maxillofac Surg.

